# Scaling India Hypertension Control Initiative strategies to 15 states—treatment outcomes and risk factors for uncontrolled blood pressure, India: a cohort study, 2018–2021

**DOI:** 10.1136/bmjopen-2025-106372

**Published:** 2025-11-28

**Authors:** Prabhdeep Kaur, Mosoniro Kriina, Parasuraman Ganeshkumar, Abhishek Kunwar, Meenakshi Sharma, Roopa Shivashankar, India Hypertension Control Initiative Collaboration, Abhinav Kadia, Balram Bhargava

**Affiliations:** 1 Isaac Centre for Public Health, Indian Institute of Science, Bengaluru, Karnataka, India; 2 ICMR - National Institute of Epidemiology, Chennai, Tamil Nadu, India; 3 World Health Organisation Country Office for India, New Delhi, Delhi, India; 4 Indian Council of Medical Research, New Delhi, Delhi, India

**Keywords:** Hypertension, Epidemiology, Cardiovascular Disease, Health policy

## Abstract

**Objectives:**

To estimate the treatment outcomes among individuals treated for hypertension in the public sector in 89 districts across 15 states in India and to identify the risk factors for uncontrolled blood pressure (BP).

**Design:**

An analysis of a cohort of people with hypertension from 2018 to 2022 from public sector health facilities.

**Setting:**

All India Hypertension Control Initiative (IHCI) implementing districts using digital information systems across 15 states of India, namely Andhra Pradesh, Bihar, Goa, Gujarat, Jharkhand, Karnataka, Maharashtra, Nagaland, Puducherry, Punjab, Rajasthan, Sikkim, Tamil Nadu, Uttar Pradesh and West Bengal.

**Participants:**

Individuals aged 30 years or older, who were diagnosed with hypertension or on medication at the time of registration between 1 January 2018 and 31 December 2021 were included in the study.

**Outcome measures:**

Treatment outcomes were controlled BP, uncontrolled BP and missed visits in the reporting quarter (1 January 2022–31 March 2022). We analysed the risk factors for uncontrolled BP.

**Results:**

Out of 1, 235, 453 hypertensive individuals enrolled in the IHCI project across 15 states, 1, 046, 512 remained under care, with 44% BP control. The control varied from 26% to 57% in various types of facilities. The states of Maharashtra, Punjab and Rajasthan had above 50% control, while Nagaland, Jharkhand and Bihar had below 25%. BP control declined from 68% when defined using a single recent reading to 52% when defined using the two-visit readings. Younger individuals (<45 years) (adjusted risk ratio, aRR2=1.05, 95% CI 1.03 to 1.06), males (aRR2=1.08, 95% CI 1.07 to 1.09), diabetics (aRR2=1.11, 95% CI 1.11 to 1.12) and those in higher-level facilities had a greater risk of uncontrolled BP.

**Conclusions:**

We documented the implementation of IHCI strategies at scale and measured treatment outcomes in a large cohort. Overall, BP control improved with variations across states. We need focused strategies to improve control in higher-level facilities, among males and people with diabetes. Using two BP readings may support consistent treatment adherence.

**STRENGTHS AND LIMITATIONS OF THIS STUDY**

- A key strength of this study is the measurement of hypertension treatment outcomes using cohort data in primary healthcare at scale in India, which has not been previously documented.
- The wide geographical and demographic representation in the dataset enhances the generalisability of the findings.
- Blood pressure measurements were based on two readings, though the interval between the two visits varied.
- We could not analyse the hypertension treatment prescriptions in this study.

## Introduction

 Hypertension, a leading cause of cardiovascular disease (CVD), represents a significant public health challenge in India.^1 2^ In 2019–2021, approximately one-fourth of individuals aged 15 and above were affected with hypertension in India, with 24.1% in men and 21.2% in women documented as per National Family Health Survey (2019–2021) data.^3^ India is a vast country and the hypertension burden varies across states, possibly due to sociodemographic and health system performance variations. The National Institution for Transforming India (NITI Aayog) developed a National Health Index (HI) score to assess the variations across states and implemented it in 2017.^4^ In 2019–2020, this score ranged from 82.2 in Kerala to 27.0 in Nagaland, highlighting differences in health system performance.^5^ For instance, Kerala, which had the highest HI score, reported 60.0% of individuals being aware and treated for hypertension, with 72.7% achieving control among those treated.^5^ In contrast, Nagaland, the state with the lowest HI score, had only 31.6% of individuals aware and treated for hypertension, with 52.0% achieving control among those treated.^5^

The World Health Organisation (WHO) framework targets include a 25% relative reduction in the prevalence of hypertension by 2025.^6^ To address this, a multipartner initiative by the Government of India, the Indian Council of Medical Research (ICMR) and the WHO launched the India Hypertension Control Initiative (IHCI) in November 2017 which aimed to reduce premature cardiovascular deaths by enhancing hypertension management and control at public sector healthcare facilities.^7^ IHCI was guided by the WHO HEARTS technical package; it emphasises evidence-based treatment guidelines, access to essential medicines and technology, patient-centred care, collaborative team-based approaches with task-sharing and robust monitoring systems. The programme was initially launched in 26 districts across five states (Punjab, Kerala, Madhya Pradesh and Maharashtra), and by 2020 it was expanded to other states.^8^

Hypertension is a major public health issue, and numerous studies have examined the factors influencing blood pressure control; however, most of these studies are based on cross-sectional community-based data,^3^ ^5^ which limits the ability to understand the risk factors in the context of cohorts on treatment in the public sector, and also temporality cannot be established. A cohort approach is essential to understand how treatment-seeking and sociodemographic factors contribute to uncontrolled BP.

The Thailand MoPH defines clinic-level BP control as systolic BP <140 mm Hg and diastolic BP <90 mm Hg at the last two consecutive visits within the fiscal year. By this definition, Thailand had controlled BP improved from 21% in 2014 to 45% in 2019, compared with 59% in 2019 when using the less stringent criterion of the last visit only.^9^ Using two visits provides a more reliable measure of sustained control and enables comparison with other countries.^10^

Hence, we analysed the data among individuals aged 30 years and above with hypertension registered under IHCI in 15 states of India from 2018 to 2021 to estimate the proportion of individuals with BP control and other treatment outcomes (uncontrolled, missed visit) by sociodemographic characteristics. We also compared the treatment outcomes by states (IHCI-implementing states) in India. We compared the proportion of BP control by one reading (recent visit BP reading) and two readings (recent visit and second last visit BP readings). We determined the risk factors of uncontrolled BP.

## Methods

### Study design and setting

The study is a secondary data analysis of a cohort of individuals with hypertension, registered from 1 January 2018 until 31 December 2021 in all primary and secondary care hospitals in the selected districts of 15 states of India (Andhra Pradesh, Bihar, Goa, Gujarat, Jharkhand, Karnataka, Maharashtra, Nagaland, Puducherry, Punjab, Rajasthan, Sikkim, Tamil Nadu, Uttar Pradesh and West Bengal) in IHCI implementing districts, which uses simple app for reporting and managing data. The IHCI programme was initiated in two phases. During the first phase, it began in Maharashtra and Punjab. In the second phase, it expanded to 13 additional states. The specific districts within these states are listed in table 1. For this study, we extracted the data from the IHCI database.

**Table 1:** Proportion registered among estimated individuals with hypertension under the India Hypertension Control Initiative, in 89 implementing districts across 15 states in India, January 2018 to December 2021

Phase	State	Districts	Month of initiation	Estimated hypertension 2021	Hypertension individuals registered (n)	Proportion of hypertension individuals registered
Phase I	Maharashtra	Bhandara, Chandrapur, Gadchiroli, Gondia, Kolhapur, Palghar, Palghar rural, Pune, Pune Municipal Corporation, Ratnagiri, Sangli, Satara, Sindhudurg, Thane, Thane rural, Wardha, Mumbai, Nagpur	January 2018	6,291,833	483,206	8
	Punjab	Amritsar, Barnala, Bathinda, Fatehgarh Sahib, Gurdaspur, Hoshiarpur, Jalandhar, Mansa, Pathankot, Rupnagar	January 2018	2,093 731	188,530	9
Phase II	Andhra Pradesh	Alluri Sitharama Raju, Anakapalli, Eluru, Krishna, NTR, Visakhapatnam	September 2020	1,181,074	27,512	2
	Bihar	Jamui, Muzaffarpur, Purnia, Rohtas, Vaishali	December 2020	1,374,316	16,194	1
	Goa	North Goa, South Goa	April 2021	223,946	19,456	9
	Gujarat	Rajkot, Surat	September 2021	1,020,079	9,824	1
	Jharkhand	Bokaro, Ranchi	February 2020	451,834	6,291	1
	Karnataka	Chikmangalur, Raichur	December 2019	314,130	30,127	10
	Nagaland	Kohima, Mokokchung	May 2021	41,431	1,240	3
	Puducherry	Puducherry	December 2020	139,711	25,993	19
	Rajasthan	Bikaner, Churu	January 2021	315,646	27,062	9
	Sikkim	East Sikkim, North Sikkim, South Sikkim, West Sikkim	July 2020	105,014	4,530	4
	Tamil Nadu	Chennai	August 2019	74,137	71,550	96
	Uttar Pradesh	Jhansi, Lalitpur, Prayagraj, Varanasi	March 2020	1,019,434	9,199	1
	West Bengal	Alipurduar, Bankura, Basirhat HD, Birbhum, Bishnupur HD, Cooch Bihar, Dakshin Dinajpur, Darjeeling, Diamond Harbour HD, Hooghly, Howrah, Jalpaiguri, Jhargram HD, Kalimpong, Kalimpong HD, Malda, Murshidabad, Nadia, Nandigram HD, North 24 Parganas, Paschim Bardhaman, Paschim Medinipur, Purba Bardhaman, Purba Medinipur, Purulia, Rampurhat HD, South 24 Parganas, Uttar Dinajpur	October 2020	8,819,390	314,739	4
	Overall			24,782,616	1,235,453	5

### Patients and public involvement

Patient and public were not directly involved in the design, conduct and dissemination plans of this study. This analysis was based on a cohort of individuals receiving care at public sector health facilities. However, it was implemented in close collaboration with government stakeholders and healthcare providers to improve hypertension care.

### Participants

Individuals 30 years or older, who had prior hypertension or were diagnosed with hypertension at the time of registration between 1 January 2018 and 31 December 2021, were included in the study. This study was based on an analysis of a large dataset. All participants were included; hence, no sample size calculation was done.

### Study population and variable definitions

*Hypertension enrolment*: People who had a diagnosis as per the state-specific protocols or were already taking antihypertensives were enrolled. Although the BP threshold for diagnosis was a systolic ≥140 mm Hg or diastolic ≥90 mm Hg, the treatment protocol differed across states.^11^ These individuals were enrolled into a simple app by a nurse and received a treatment card containing a unique identification code and a QR code, which facilitated follow-up visits for longitudinal data collection.

*Under care*: Individuals registered from 1st January 2018 until 31st December 2021 and came for follow-up at least once a year from 1st April 2021 to 31st March 2022.

*Reporting quarter:* Individuals’ most recent visit between 1 January 2022 and 31 March 2022 was taken as the reporting quarter.

*Controlled BP*: Individuals who had BP systolic <140 mm Hg and BP diastolic <90 mm Hg during the most recent visit in the reporting quarter.

*Uncontrolled BP*: Individuals with BP systolic ≥140 mm Hg or BP diastolic ≥90 mm Hg during the most recent visit in the reporting quarter.

*Missed visit*: Individuals who did not come for the follow-up to the facility even once in the reporting quarter, among individuals registered before 31 December 2021. This category also includes individuals who have visited but did not measure their BP.

*Lost to follow-up*: Registered individuals who did not come for follow-up even once from 1st April 2021 to 31st March 2022 or have died.

*BP control based on two readings:* Individuals who had BP systolic <140 mm Hg and BP diastolic <90 mm Hg on both occasions of ‘recent visit’ and ‘second last visit’.

#### Outcome variables

1. Hypertension status, categorised as controlled or uncontrolled BP, based on the most recent visit of the reporting quarter, was considered one of the outcome variables. Uncontrolled BP was the primary outcome of interest.
2. Hypertension status, determined by two BP readings, was also included as another outcome variable.

#### Exposure variable

We analysed various risk factors like age which were categorised into three groups (<45, 45–54, >55), gender (male, female) and facilities were classified as general hospitals (GH), offering broad inpatient and specialist services; district hospitals (DH), serving as secondary referral centres for the district; sub-district hospitals (SDH), first referral units with 30–100 beds; community health centres (CHC), block-level facilities with limited specialist care; primary health centres (PHC), providing basic outpatient and preventive services; and health and wellness centres/sub-centres (HWC/SC), delivering primary preventive and promotive care at the community level.^12^ These factors also included prior diabetes mellitus (DM), which was categorised as ‘yes’ for individuals who had a history of diabetes or were diagnosed by a physician for diabetes. Prior CVD was categorised as ‘yes’ for those individuals who had a prior stroke or prior heart attack. Individuals taking hypertension medicine at registration were categorised as ‘yes’, else they were categorised as ‘no’.

### Data management and analysis

We analysed data from all IHCI-implementing facilities where patient details were entered through a hand-held mobile device application called Simple.^13^ Simple was designed to quickly and accurately register patients, record BPs over time and record medication regimens. De-identified data were extracted using Metabase, an open-source data visualisation and analytics platform.^14^ Data cleaning, coding and analysis were done in STATA SE (V.17.0) software (StataCorp, Texas, USA). Categorical variables were expressed as frequency and proportion, while continuous variables were reported using the median and IQR.

### Descriptives

1. *Proportion of individuals registered to IHCI*: We computed the proportion of registered individuals with hypertension in IHCI by using the estimated hypertension individuals as the denominator. We estimated this individual by using census data for 2001 and 2011 for the districts in 15 states under IHCI.
2. *Treatment outcome:* We analysed the proportion of treatment outcomes among the under care by demographic and biological characteristics. Treatment outcomes included controlled BP, uncontrolled BP and missed visits.
3. *Comparison of treatment outcome by state*: Treatment outcomes were estimated and compared by state in proportions.
4. *Comparison of one and two readings BP control:* To compare the proportion based on one and two readings, individuals whose BP was not taken in the reporting quarter were excluded. Thus, this consists of individuals whose BP was either controlled or uncontrolled only.

### Analytical

*Risk factors of uncontrolled BP:* To determine the risk factors of uncontrolled BP based on the most recent visit of the reporting quarter, we excluded individuals whose BP readings were not taken in the first quarter of 2022. Our analysis focused solely on individuals with controlled and uncontrolled BP as the dependent variable. The risk factors of uncontrolled hypertension were identified using a generalised linear model (GLM). GLM univariate regression was used with age, gender, facility type, prior diabetes, prior CVD and taking a hypertension drug at registration as exposure variables, to calculate the unadjusted risk ratio (RR). After checking for multicollinearity using the variation inflation factor, GLM multivariable regression was used to calculate the adjusted risk ratio (aRR) by adjusting for all variables in the univariate analysis. The results were presented in terms of RR, aRR, 95% CI and p value. P value below 0.05 was considered statistically significant. A similar analysis was performed to identify risk factors of uncontrolled BP based on two readings.

## Results

We enrolled 1 235 453 individuals with hypertension in the project districts in 15 states, contributing to about 5% of the estimated hypertension in project districts in these states (table 1). This proportion was highest in Tamil Nadu (96%), followed by Puducherry (19%) and lowest in Rajasthan (1%). Of the enrolled individuals, 188 941 (15%) were either lost to follow-up or died. Hence, 1 046 512 were under care during the reporting quarter. All the analyses in this paper are based on 1 046 512 people with hypertension under care. The majority (722 994, 69%) of the individuals under care belong to the ≥55 years age group (online supplemental table 1), with a median age of 61 years (IQR: 54–67 years). The proportion of females was higher (630 852, 60%) as compared with males. The majority of the individuals were registered in HWC/SC (38%), and the least (11%) were in GH/DH/SDH. The proportion of individuals with diagnosed diabetes among under-care individuals was 26% (769 967) and 2% (20 976) individuals reported prior CVD. Among the individuals under care, 41% (430 709) were already taking hypertension drugs at the time of registration. The median follow-up months from the date of registration to the most recent visit was 6 months (IQR=11 months). The median interval between two BP measurements was 40 days (IQR: 30–82), and in all states, the median gap was 1–2 months (online supplemental table 2).

### Treatment outcomes by sociodemographic characteristics

Overall BP control among the individuals under care was 44% (457,314), 20% (378,802) had uncontrolled BP, and 36% (1,046,512) missed visits (table 2). Uncontrolled BP in the facility was lowest in HWC/SC (15%, 60,496), followed by PHC 22% (83,255), GH/DH/SDH 24% (26,280) and the highest in CHC with 26% (40,365). Among individuals who had prior DM, 40% (109 919) had their BP under control and individuals with prior CVD had BP control of 43% (9,107). Among those individuals who were already on hypertension drugs at the time of registration, 39% (1,69,553) had their BP under control.

**Table 2:** Treatment outcomes among individuals with hypertension under care by sociodemographic characteristics under India Hypertension Control Initiative in the reporting quarter in 15 states of India, January–March 2022 (N=1,046,512)

Characteristics	Categories	Controlled BP		Uncontrolled BP		Missed visit		Total
		n	%	n	%	n	%	n
	Overall	457,314	44	210,396	20	3,78 802	36	1,046,512
Age	<45	36,324	39	17,436	19	39,400	42	93,160
	45–54	97,141	42	46,487	20	86,730	38	230,358
	≥55	323,849	45	146,473	20	252,672	35	722,994
Gender	Female	282,585	45	121,395	19	226,872	36	630,852
	Male	174,729	42	89,001	21	151,930	37	415,660
Type for facility	GH/DH/SDH	38,219	35	26,280	24	46,194	42	110,693
	CHC	41,191	26	40,365	26	75,448	48	157,004
	PHC	149,799	39	83,255	22	148,432	39	381,486
	HWC/SC	228,105	57	60,496	15	108,728	27	397,329
Diabetes mellitus	No	347,395	45	142,841	19	279,731	36	769,967
	Yes	109,919	40	67,555	24	99,071	36	276,545
Prior CVD	No	448,207	44	205,672	20	371,657	36	1,025,536
	Yes	9,107	43	4,724	23	7,145	34	20,976
Taking hypertension drug at registration	No	287,761	47	107,491	17	220,551	36	615,803
	Yes	169,553	39	102,905	24	158,251	37	430,709
Phase	Phase I	313,498	46	127,276	19	243,605	36	684,379
	Phase II	143,816	40	83,120	23	135,197	37	362,133

BP, blood pressure; CHC, community health centres; CVD, cardiovascular disease; GH/DH/SDH, general hospitals, district hospitals and sub-district hospitals; HW/SC, health wellness centre/sub-centre; PHC, primary health centre.

### Treatment outcomes by state

**Figure 1:**
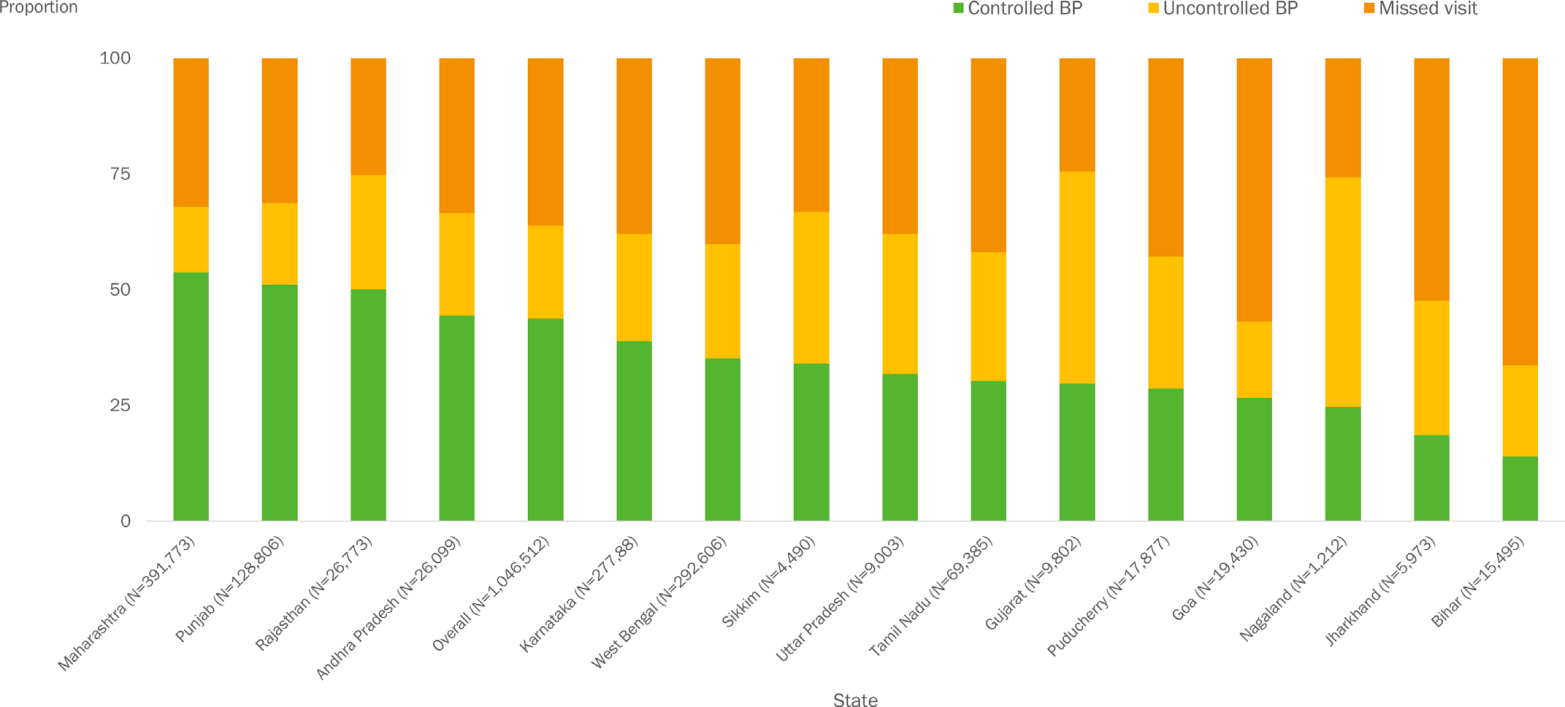
Treatment outcome among individuals with hypertension undercare by state under India Hypertension Control Initiativein the reporting quarter in 15 states of India, January–March 2022 (N=1,046,512).

While comparing the treatment outcomes by states, the proportion of controlled BP was highest in Maharashtra (54%; 210,467), followed by Punjab (51%; 65,857), and lowest in Bihar (14%; 2,164), followed by Jharkhand (19%; 1,112) (figure 1). The proportion of uncontrolled BP was highest in Nagaland (50%; 602) and lowest in Maharashtra (14%; 55,278). The proportion of missed visits was highest in Bihar (66%; 10,286) and lowest in Gujarat (24%; 2,400).

### Treatment outcome based on one and two readings

**Figure 2:**
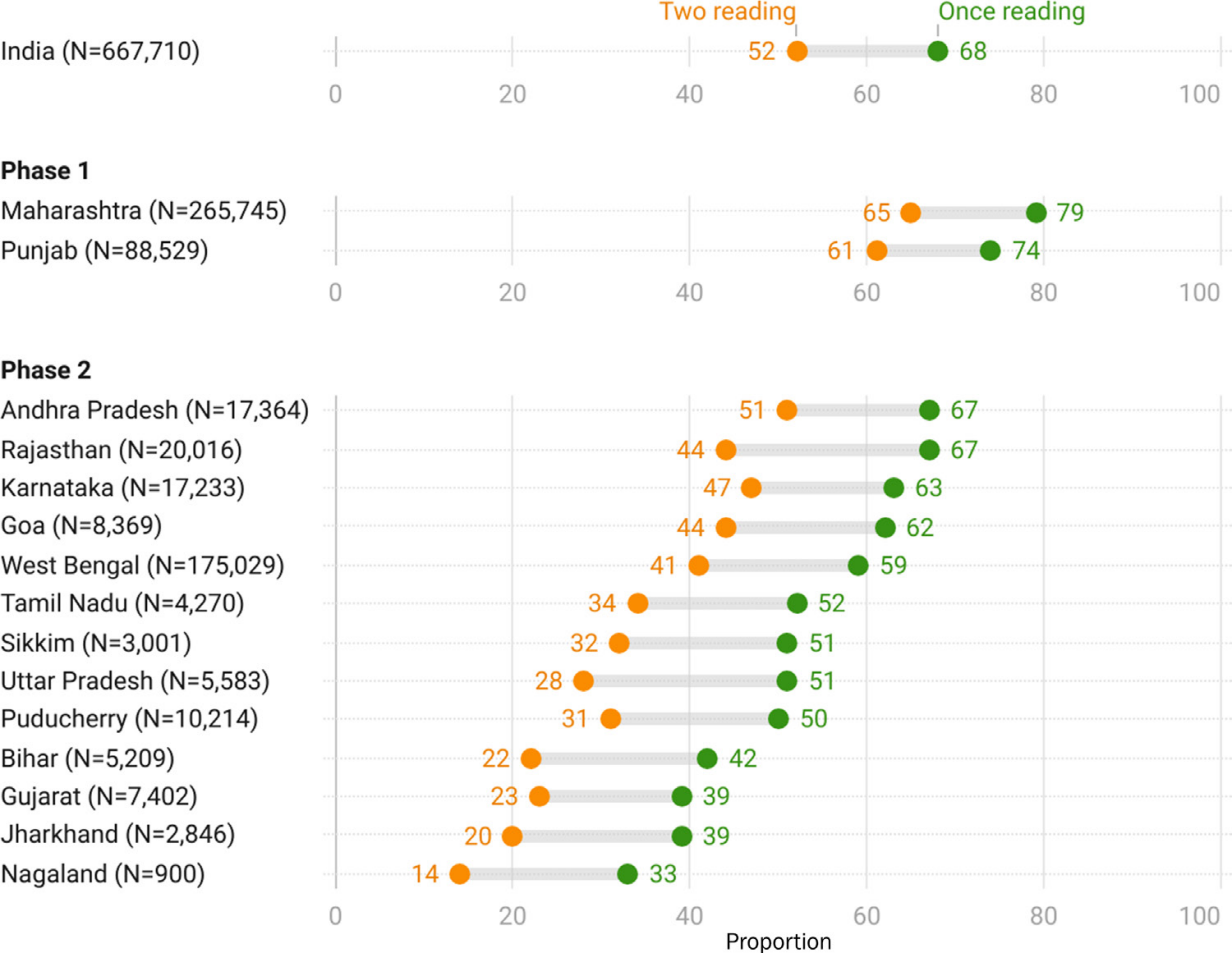
Blood pressure control based on two readings among individuals with hypertension undercare by the state in India Hypertension Control Initiative (IHCI) in the reporting quarter in 15 states of India, January–March 2022 (N=667,710).

We also estimated BP control using readings from two separate visits: the ‘recent visit’ and the ‘second-last visit’, considering only individuals who had their BP measured on both occasions (two readings) (figure 2). Of the 1,046,512 undercare, 667,710 (64%) had two readings. The median duration of follow-up from the second last visit to the most recent visit was 1 month (IQR=1 month). Overall, the proportion of controlled BP based on one reading was 68% (457,314) and 52% (350,347) for two readings. The difference in proportion under control while comparing two versus one reading for phase I states Punjab (61% vs 74%) and Maharashtra (65% vs 79%) was 13%–14%. In other states it varied from 16% to 23%.

### Risk factors of uncontrolled BP

We analysed the risk factors for uncontrolled BP among 667 710 who reported a visit between January and March 2022 (online supplemental table S3, table 3). Based on two blood pressure readings, individuals aged 45–54 years had a higher risk of uncontrolled BP compared with those aged ≥55 years (table 3). Males had a higher risk of uncontrolled BP compared with females (aRR2=1.08, 95% CI 1.07 to 1.09). Additionally, individuals with DM exhibited a higher risk (aRR2=1.11, 95% CI 1.11 to 1.2). Those taking hypertension drugs at the time of registration also had a higher risk of having uncontrolled BP (aRR2=1.18, 95% CI 1.17 to 1.19) as compared with those who were newly initiated on treatment at registration. Regarding facility type, using HWC/SC as the reference category, all other health facilities had a higher risk of having uncontrolled BP. Similar risk factors were observed when considering one BP reading for the outcome variable of uncontrolled BP.

**Table 3:** Predictors of uncontrolled BP among individuals with hypertension undercare under India Hypertension Control Initiative in the reporting quarter in 15 states of India, January–March 2022 (N=667,710)

Characteristics	Categories	Uncontrolled BP (one reading)				Uncontrolled BP (two readings)			
		n (%)	aRR1*	95% CI		n (%)	aRR2*	95% CI	
Age	≥55	146,473 (31)	Ref.			221,010 (47)	Ref.		
	45–54	46,471 (32)	1.03	1.02	1.04	69,765 (49)	1.03	1.02	1.03
	<45	17,425 (32)	1.04	1.03	1.06	26,388 (49)	1.05	1.03	1.06
Gender	Female	121,369 (30)	Ref.			184,636 (46)	Ref.		
	Male	88,945 (34)	1.09	1.08	1.10	132,527 (50)	1.08	1.07	1.09
Diagnosed diabetes	No	142,782 (29)	Ref.			220,489 (45)	Ref.		
	Yes	67,532 (38)	1.17	1.16	1.18	96,674 (54)	1.11	1.11	1.12
Prior CVD	No	205,672 (31)	Ref.			310,280 (47)	Ref.		
	Yes	4,724 (34)	0.98	0.95	1.01	6,883 (50)	0.97	0.95	1.00
Taking hypertension drug at registration	No	107,452 (27)	Ref.			166,946 (42)	Ref.		
	Yes	102,862 (38)	1.22	1.21	1.23	150,217 (55)	1.18	1.17	1.19
Facility type	HWC/SC	60,472 (21)	Ref.			100,165 (35)	Ref.		
	PHC	83,225 (36)	1.58	1.56	1.60	123,921 (53)	1.45	1.43	1.46
	GH/DH/SDH	26,267 (41)	1.83	1.80	1.86	36,948 (57)	1.58	1.56	1.60
	CHC	40,350 (49)	2.18	2.15	2.21	56,129 (69)	1.86	1.84	1.88
Phase	Phase I	127,276 (29)	Ref.			196,467 (45)	Ref.		
	Phase II	83,120 (37)	1.18	1.16	1.19	120,696 (53)	1.12	1.11	1.13

* aRR1 and aRR2, the models are adjusted for all variables like age, gender, diagnosed diabetes, prior cardiovascular disease (CVD), taking medicine at registration, facility type and phase of the study.

aRR, adjusted risk ratio; BP, blood pressure; CHC, community health centres; GH/DH/SDH, general hospitals, district hospitals and sub-district hospitals; HWC/SC, health wellness centre/sub-centre; PHC, primary health centre.

## Discussion

IHCI strategies were scalable in diverse health systems across 15 Indian states in 89 districts in a relatively short period. We documented the early outcomes in 26 districts in 2018–2020 and demonstrated the scalability of five core strategies to improve BP control.^8^ In the scale-up phase, despite rapid expansion and minimal support from the project team, nearly half of the enrolled people with hypertension are under care at public sector health facilities. This indicated the integration of strategies in the routine healthcare system.

The project was expanded in a phased manner between 2018 and 2022. Based on one visit BP reading (reporting quarter), the study districts achieved 44% BP control among undercare individuals. As per the recent study which used national-level survey NFHS-5 data conducted in 2019–2021, the control among people with hypertension on medications (including people on treatment in the public or private sector) ranged from 52% to 85% in states.^5^ The project districts in Maharashtra and Punjab achieved more than 50% control among people on treatment in our study. Our results were consistent with the NFHS-5, which also reported high control among treated in Maharashtra (77%) and Punjab (68%).^5^ We observed low BP control in states such as Bihar, Jharkhand and Nagaland, consistent with data from the national survey. According to NFHS-5, the prevalence of hypertension was 27.9% in Bihar, 30.1% in Jharkhand and 29.8% in Nagaland, which is higher than the overall prevalence in India (28.1%).^15^ However, among those diagnosed with hypertension, 61.4% in Bihar, 53.4% in Jharkhand and 66.0% in Nagaland remained untreated, and uncontrolled BP among treated was 52.3% in Bihar, 62.1% in Jharkhand and 83.9% in Nagaland.^15^ The BP control may also be influenced by the overall capacity of the public health system. NITI AAYOG, India’s policy think tank, devised HI scores to stratify the state’s capacity based on various health indicators.^4^ The poorly performing states in our study also had low HI. Overall, the states with poor health indices are yet to shift their focus from infectious diseases/maternal child health programmes to non-communicable diseases (NCDs) at the policy and planning level. Hence, the stakeholders need to be sensitised regarding the need for prioritising NCDs. At the level of implementation, possible reasons for poor hypertension control are low awareness, inadequate primary care infrastructure, trained human resources and unreliable availability of drugs. The interventions to strengthen primary care will include a trained workforce for community and opportunistic screening, well-equipped primary care facilities with medications/BP monitors and community awareness programmes. We recommend that NITI AAYOG HI may consider BP control as one of the key indicators for UHC to nudge the states to focus on NCDs. Our study analysed the data when the project was rapidly expanding across a large number of districts with minimal additional resources; quality improvement initiatives will be required to improve retention in care and control once all the key strategies are in place in the phase II districts.

We reported the hypertension control based on the recent visit in the reported quarter as per the WHO HEARTS recommended indicator.^16^ We also explored the change in control if two consecutive BP readings were considered among controlled and uncontrolled individuals. The overall control was reduced by 16% if two visits were considered. Overall, BP control based on the recent visit is a useful indicator and easy to measure to track the performance of health facilities or districts over time. However, the use of two consecutive readings for BP control can be used as a quality indicator for hypertension programmes. The control among treated enables comparisons across states with India and with other countries implementing the HEARTS package. A WHO report on implementing the HEARTS package reported treatment of over 17.4 million people with hypertension^17^ and reported BP control indicators based on the data from health facilities in various settings.^18^ Thailand has nearly 1.7 million people with hypertension enrolled between January 2018 and June 2020, and 59% had controlled BP in a recent visit.^18^ In 2020, a few countries with more than 50000 patients enrolled and high BP control include Turkey 59% (N=152,155), Cuba 67% (N=65,832) and Vietnam 79% (N=70,586).^18^ In our setting, two phase I states, Punjab (N=88,529) and Maharashtra (N=265,467), achieved above 60% control in both one and two readings even with rapid scaling of the programme

The risk of uncontrolled BP was higher among males and people with diabetes in our study. Our observation was consistent with the findings from a community-based national survey conducted in 2019–2021, which reported 1.7 (95% CI 1.21 to 2.41) times higher odds of controlled BP among women than men.^5^ The odds of controlled BP among diabetics were lower (OR 0.79 (95% CI 0.63 to 0.99)) when compared with those with no diabetes.^5^ The higher uncontrolled BP among males in facility-based surveys was also documented in low- and middle-income countries such as Ethiopia, Ghana and Thailand.^19^,^21^ A survey of 65 667 individuals with hypertension visiting both public and private hospitals in Bangkok, Thailand estimated higher odds (OR 1.16, 95% CI 1.10 to 1.20) of uncontrolled BP among males compared with females and among people with diabetes (1.16, 95% CI 1.08 to 1.23) than those without diabetes.^21^ The reasons for higher controlled BP among males could be explored further. Possible hypotheses include other risk factors such as smoking or alcohol use. There is ample evidence that the combination of hypertension and diabetes amplifies the cardiovascular risk, hence the need for tighter BP control.^5 21^ We observed poor control among young people with hypertension below 45 years. Our findings align with the National Health and Nutrition Examination Survey 2017–2020 conducted in the USA among 3954 hypertensive individuals, which reported a higher prevalence of uncontrolled hypertension among adults aged 18–44 years (93.4%, 95% CI 90.3% to 96.4%) compared with those aged 65 years or older (69.7%, 95% CI 66.7% to 72.7%). ^22^ Poor BP control in younger individuals poses a major risk for cardiovascular events as reported in a systematic review including seventeen observational cohorts of 4,533,292 young adults. There was an increase in cardiovascular risk across BP categories, namely grade 1 hypertension (1.92, 95% CI 1.68 to 2.19), to grade 2 hypertension (3.15, 95% CI 2.31 to 4.29), compared with optimal BP.^23^ Younger hypertensive individuals were reported to have higher rates of cigarette use, obesity, dyslipidaemia and excessive salt consumption than the general population.^24^ Centers for Disease Control and Prevention guidelines suggest eating a healthy diet, being physically active, not smoking, limiting the use of alcohol and getting enough sleep to prevent and control blood pressure.^25^ The evidence suggests the need for patient education and focused strategies to monitor BP control among males and people with diabetes.

We observed a 1.5 to two times higher risk of uncontrolled BP at the district level and block-level health facilities (CHC) compared with Health and Wellness Centres, which catered to a few villages. In the early phase of the project, we observed 42% control in Health and Wellness Centres in 26 phase I districts compared with 35% in the district hospitals.^8^ The pattern consistency, even after the project was expanded from five to more than 15 states, suggests the need to improve hypertension care by linking the patients seeking care in the high-level facilities to HWC catering to their area. The possible reasons for high uncontrolled BP at higher level facilities might be the likelihood of complex cases which are being referred and the inability to recall the patients who did not visit regularly due to lack of outreach community health workers. The higher odds of uncontrolled BP at advanced-level health facilities were also documented in Thailand. A survey of 65,667 individuals with hypertension visiting both public and private hospitals in Bangkok, Thailand, estimated 1.23 (95% CI 1.16 to 1.30) times higher odds of uncontrolled BP at the middle level and 1.12 (95% CI 1.03 to 1.22) times higher odds at the advanced level health facilities.^21^ An example of high BP control with the implementation of primary care-based interventions is the population served by Kaiser Permanente in Northern California. The interventions such as protocol-based management, quality improvement initiatives and a robust digital monitoring system improved the BP control from 45% to 90% in a cohort of 650,000 people over 13 years (2000–2013).^26^ A study in the primary care clinics from Greenville, USA implemented an intervention in 16 sites enrolling 21 611 people and documented improvement in the BP control from 64% at the baseline to 74% at 1 year.^27^ The possible reasons for high control in the HWC could be proximity to patients’ homes, personalised interaction with the nurses and community healthcare workers (5000 population catered by HWC), easy to recall patients if they miss refills and no costs for travel and medications.^12 28^ Primary care-based hypertension management has consistently shown better patient outcomes across HIC and LMIC, calling for investments in the primary care for NCDs.

In this manuscript, we have reported data from public sector facilities. However, in India, a large proportion of people with hypertension seek care in the private sector. As per estimates from the 75th round of National Sample Survey Office 2018, the private sector accounted for 53.8% of hypertension-related hospitalisations and 61.2% of outpatient visits.^29^ IHCI community surveys conducted in 2018–2019 and 2023–2024 across five states of India reported BP control improved in both sectors, increasing from 37% to 38% in government facilities and 35% to 47% in private facilities.^30^ Over the same period, care-seeking in the private sector declined from 67.5% to 60.2%, while use of government facilities rose, especially at HWC (1%–3.1%) and PHCs/CHCs (10%–20.1%).^30^ These findings indicate a gradual strengthening of public sector hypertension services and growing community trust in government health facilities over time. The best practices in the public sector can also be disseminated to the private sector to improve hypertension control.

### Limitations

When interpreting this study’s results, there are a few limitations to be considered. First, in the case of BP readings considered for ‘two readings’, the interval between the two visits was variable. Additionally, our research did not include an analysis of prescriptions. Our team has analysed the prescriptions in two of the states, and they have been published.^31^ Though all states used a simple app for data entry, there could have been differences in the quality of data recording across states. Although some patients had a missed visit in the reporting quarter, which could bias the results, the age and gender distribution were similar among those with and without missed visits (online supplemental table S4). Our study enrolled only ~5% of the estimated hypertensive population. However, it represents at least one third of the treated because the national treatment coverage in India was 14.5% in 2017–2018.^32^

### Conclusions

We demonstrated a scalable model of hypertension management in primary care across diverse health systems in India. All states demonstrated improvement in BP control among people treated in the public sector, although the control varied depending on the ability of the health system to integrate various processes effectively. The continuum of care documented the consistent BP control across two visits in a large proportion of people treated in the project districts. The high control in the HWC and PHC was encouraging and built a strong evidence base for scaling and strengthening hypertension treatment closer to patients’ homes. We recommend scaling the cohort monitoring using simple digital tools and capacity building of healthcare workers to manage hypertension in primary care settings. The monitoring systems need to be robust, to enable healthcare workers to track retention in care and control. The BP control can be monitored using two BP readings if feasible for assessing consistent adherence to treatment.

## Supplementary material

10.1136/bmjopen-2025-106372 online supplemental file 1

## Data Availability

Data are available upon reasonable request.
