# Supplementary material for: Scaling India Hypertension Control Initiative strategies to 15 states—treatment outcomes and risk factors for uncontrolled blood pressure, India: a cohort study, 2018–2021
Source: BMJ Open. 2025 Nov 28;15(11):e106372. doi: 10.1136/bmjopen-2025-106372 (PMC12666080; doi:10.1136/bmjopen-2025-106372)
Supplement: online supplemental file 1 [file bmjopen-15-11-s001.docx]

**Supplementary Table 1: Sociodemographic characteristics of individuals with hypertension under IHCI in the reporting quarter in 15 states of India, January-March 2022**

|  |  | **Undercare (N=1,046,512)** | | **BP value available at reporting quarter (N=667,710)** | |
| --- | --- | --- | --- | --- | --- |
| **Characteristics** | **Categories** | **n** | **%** | **n** | **%** |
| **Age** | <45 | 93,160 | 9 | 53,760 | 8 |
|  | 45-54 | 230,358 | 22 | 143,628 | 22 |
|  | >=55 | 722,994 | 69 | 470,322 | 70 |
| **Gender** | Female | 630,852 | 60 | 403,980 | 61 |
|  | Male | 415,660 | 40 | 263,730 | 40 |
| **Diagnosed Diabetes** | No | 769,967 | 74 | 490,236 | 73 |
|  | Yes | 276,545 | 26 | 177,474 | 27 |
| **Prior CVD** | No | 1,025,536 | 98 | 653,879 | 98 |
|  | Yes | 20,976 | 2 | 13,831 | 2 |
| **Taking hypertension drug at registration** | No | 615,803 | 59 | 395,252 | 59 |
|  | Yes | 430,709 | 41 | 272,458 | 41 |
| **Facility type**^*^ | GH/DH/SDH | 110,693 | 11 | 64,499 | 10 |
|  | CHC | 157,004 | 15 | 81,556 | 12 |
|  | PHC | 381,486 | 36 | 233,054 | 35 |
|  | HWC/SC | 397,329 | 38 | 288,601 | 43 |
|  | Total | 1,046,512 | 100 | 667,710 | 100 |

Facility type^*^- HWC/SC: health wellness centre/sub-centre, PHC: primary health centre, CHC: community health centres, GH/DH/SDH: general hospitals, district hospitals, and sub-district hospital

**Supplementary Table 2: Time Interval in days between the two readings (recent and second last visit) among individuals with Blood pressure reading under IHCI in the reporting quarter in 15 states of India, January-March 2022**

| **States** | **n** | **Median** | **Quartile 1** | **Quartile 3** |
| --- | --- | --- | --- | --- |
| Andhra Pradesh | 26,099 | 35 | 30 | 72 |
| Bihar | 15,495 | 42 | 30 | 71 |
| Goa | 19,430 | 56 | 31 | 91 |
| Gujarat | 9,802 | 35 | 29 | 60 |
| Jharkhand | 5,973 | 42 | 28 | 82 |
| Karnataka | 27,788 | 43 | 30 | 77 |
| Maharashtra | 391,773 | 46 | 31 | 100 |
| Nagaland | 1,212 | 35 | 29 | 61.5 |
| Puducherry | 17,877 | 62 | 32 | 105 |
| Punjab | 128,806 | 44 | 30 | 110 |
| Rajasthan | 26,773 | 34 | 29 | 52 |
| Sikkim | 4,490 | 42 | 31 | 68 |
| Tamil Nadu | 69,385 | 44 | 30 | 86 |
| Uttar Pradesh | 9,003 | 33 | 29 | 49 |
| West Bengal | 292,606 | 34 | 28 | 56 |
| **Total** | **1,046,512** | **40** | **30** | **82** |

**Supplementary Table 3: Predictors of uncontrolled BP among individuals with hypertension undercare, under IHCI in the reporting quarter in 15 states of India, January-March 2022 (N=667,710)**

|  |  | **Uncontrolled BP (One reading)** | | | | **Uncontrolled BP (Two readings)** | | | |
| --- | --- | --- | --- | --- | --- | --- | --- | --- | --- |
| **Characteristics** | **Categories** | **n (%)** | **RR1*** | **95% CI** | | **n (%)** | **RR2†** | **95% CI** | |
| **Age group**  **(in years)** | ≥55 | 146,473 (31) | ref. |  |  | 221,010 (47) | ref. |  |  |
|  | 45-54 | 46,471(32) | 1.04 | 1.03 | 1.05 | 69,765 (49) | 1.04 | 1.04 | 1.05 |
|  | <45 | 17,425(32) | 1.04 | 1.03 | 1.05 | 26,388 (49) | 1.04 | 1.03 | 1.04 |
| **Gender** | Female | 121,369(30) | ref. |  |  | 184,636 (46) | ref. |  |  |
|  | Male | 88,945(34) | 1.12 | 1.12 | 1.13 | 132,527 (50) | 1.10 | 1.09 | 1.11 |
| **Diagnosed Diabetes** | No | 142,782(29) | ref. |  |  | 220,489 (45) | ref. |  |  |
|  | Yes | 67,532(38) | 1.31 | 1.30 | 1.32 | 96,674 (54) | 1.21 | 1.20 | 1.22 |
| **Prior CVD** | No | 205,672 (31) | ref. |  |  | 310,280 (47) | ref. |  |  |
|  | Yes | 4724 (34) | 1.09 | 1.10 | 1.18 | 6,883 (50) | 1.05 | 1.03 | 1.07 |
| **Taking hypertension drug at registration** | No | 107,452(27) | ref. |  |  | 166,946 (42) | ref. |  |  |
|  | Yes | 102,862(38) | 1.39 | 1.38 | 1.40 | 150,217 (55) | 1.31 | 1.30 | 1.31 |
| **Facility type** | HWC/SC | 60,472(21) | ref. |  |  | 100,165 (35) | ref. |  |  |
|  | PHC | 83,225(36) | 1.70 | 1.69 | 1.72 | 123,921 (53) | 1.53 | 1.52 | 1.54 |
|  | GH/DH/SDH | 26,267(41) | 1.94 | 1.92 | 1.97 | 36,948 (57) | 1.65 | 1.64 | 1.66 |
|  | CHC | 40,350(49) | 2.36 | 2.34 | 2.38 | 56,129 (69) | 1.98 | 1.97 | 2.00 |
| **Phase** | Phase I | 127,276(29) | ref. |  |  | 196,467 (45) | ref. |  |  |
|  | Phase II | 83,120(37) | 1.27 | 1.26 | 1.28 | 120,696 (53) | 1.19 | 1.19 | 1.20 |

Facility type^*^- HWC/SC: health wellness centre/sub-centre, PHC: primary health centre, CHC: community health centres, GH/DH/SDH: general hospitals, district hospitals, and sub-district hospitals. RR1† unadjusted risk ratio based on one BP reading & RR2‡ unadjusted risk ratio based on two BP reading

**Supplementary Table 4: Descriptives of age and gender among individuals with hypertension undercare, under IHCI in the reporting quarter in 15 states of India, January-March 2022**

| **Variable** | **Category** | **Visited with BP** | | **Missed visit** | |
| --- | --- | --- | --- | --- | --- |
|  |  | n | % | n | % |
| Age | <45 | 53,760 | 8 | 39,400 | 10 |
|  | 45-54 | 1,43,628 | 22 | 86,730 | 23 |
|  | >=55 | 4,70,322 | 70 | 2,52,672 | 67 |
| Gender | Female | 4,03,980 | 61 | 2,26,872 | 60 |
|  | Male | 2,63,730 | 39 | 1,51,930 | 40 |
|  | Total | 6,67,710 | 100 | 3,78,802 | 100 |
