# Supplementary material for: Scaling India Hypertension Control Initiative strategies to 15 states—treatment outcomes and risk factors for uncontrolled blood pressure, India: a cohort study, 2018–2021
Source: BMJ Open. 2025 Nov 28;15(11):e106372. doi: 10.1136/bmjopen-2025-106372 (PMC12666080; doi:10.1136/bmjopen-2025-106372)
Supplement: Supplementary file 2 [file bmjopen-2025-106372.reviewer_comments.pdf]

PEER REVIEW HISTORY

BMJ Open publishes all reviews undertaken for accepted manuscripts. Reviewers are asked to complete a checklist review form and are provided with free text boxes to elaborate on their assessment. These free text comments are reproduced below.

ARTICLE DETAILS

Title (Provisional)

Scaling India Hypertension Control Initiative strategies to 15 states - Treatment Outcomes and Risk Factors for Uncontrolled Blood Pressure, India – A cohort study, 2018–2021

Authors

Kaur, Prabhdeep; Kriina, Mosoniro; Ganeshkumar, Parasuraman; Kunwar, Abhishek; Sharma , Meenakshi; Shivashankar, Roopa; Hypertension Control Initiative Collaboration, India; Bhargava, Balram

VERSION 1 - REVIEW

**Reviewer**	**1**
**Name**	**Renna, Nicolas**
**Affiliation**	**Hospital Español de Mendoza , Department of Cardiology**
**Date**	**16-Aug-2025**
**COI**	**None**

Strengths

This is the largest cohort-based assessment of hypertension treatment outcomes in the Indian public health system, covering 15 states and over 1.2 million patients.

Clear demonstration of IHCI scalability, with strong real-world relevance.

Rigorous use of standardized definitions (controlled/uncontrolled BP, missed visits).

The comparison between one vs. two BP readings is a valuable methodological insight with direct programmatic implications.

Points for improvement

Abstract: Consider explicitly stating that BP control fell from 68% (one reading) to 52% (two readings), as this is an important message for program monitoring.

Methods:

Clarify the interval between the two BP readings and whether there was heterogeneity across states/facilities.

Specify if missing data handling (e.g., patients without BP measurement in the quarter) could bias the results.

Results:

While state-level outcomes are shown, a more explicit link with NITI Aayog Health Index scores in the Results section (not only Discussion) would enrich interpretation.

Figures: The bar charts are informative, but the y-axis labels in Figure 1 are somewhat cluttered; consider simplifying for clarity.

Discussion:

The finding of higher uncontrolled BP in higher-level facilities deserves more explanation—whether due to referral of more severe/complex cases or system-level issues.

Consider briefly comparing with private sector control rates, as many hypertensives in India seek care outside the public system.

Expand on policy implications: how can IHCI bridge state disparities, especially in Bihar, Jharkhand, Nagaland?

Limitations:

Prescription data absence is acknowledged, but variability in digital recording/reporting quality across states should also be noted.

Selection bias: only 5% of estimated hypertensives were enrolled—this may limit generalizability.

Language/Style

The manuscript is well written; a few long sentences could be shortened for readability.

**Reviewer**	**2**
**Name**	**Goto, Kenichi**
**Affiliation**	**Kyushu University, Health Sciences**
**Date**	**25-Aug-2025**
**COI**	**None**

General comments:

This paper explores the status of blood pressure control and the factors contributing to poor control across 15 states in India, utilizing large-scale data. It offers valuable insights for hypertension management in India. The reviewer provides the following comments:

Major:

1. Page 5, Line 103: It is stated that hypertension was diagnosed according to state-specific protocols, but do the diagnostic criteria for hypertension actually differ by state? Aren't they standardized as 140/90 mmHg or above? If they are not standardized at 140/90 mmHg, please specify the diagnostic criteria for hypertension for each state.

2. Page 7, Line 140 and Limitations: Kindly provide a description of what 'Metabase' is and what its primary functions are. Is this application the default option used during visits to medical facilities in India? If not, it is possible that individuals who use digital devices are more health-conscious, which may introduce a potential healthy-user bias. Please consider and discuss this point in the Limitations section.

3. Page 17, Paragraph 2: As shown in Figure 1, treatment outcomes among individuals with hypertension vary considerably across states. What are the potential reasons for these disparities? Furthermore, what policy interventions or strategies could be effective in narrowing these gaps? A more in-depth discussion on these issues is encouraged.

4. Page 19, Paragraph 4: In Table 3, younger age is also identified as a significant factor associated with uncontrolled blood pressure. However, this aspect has not been addressed in the Discussion. Poor blood pressure control in younger individuals poses a major risk for the development of cardiovascular complications. Please also discuss the possible reasons for poor blood pressure control among younger adults, as well as potential strategies to address this issue.

5. Page 20, paragraph 5: What factors might contribute to better blood pressure control among patients in HWC/SC settings? For instance, could patient education play a significant role? This point should be further discussed.

Minor:

1. Page 4, Lines 71-72: The sentences are duplicated. Please delete them.

2. Page 4, Lines 77-79: Please briefly explain the rationale for comparing blood pressure control rates based on a single measurement versus two repeated measurements.

3. Page 6, Lines131-132: Please provide the definitions of General Hospital, District Hospital, and Sub-District Hospital in India.

4. Page 9, Line 184: Kindly include the average age of all subjects.

VERSION 1 - AUTHOR RESPONSE

	Reviewers	Comments to the Author:	Revision	Page no.	line no.

**Reviewer 1**	Comment 1	Abstract: Consider explicitly stating that BP control fell from 68% (one reading) to 52% (two readings), as this is an important message for program monitoring.	We thank the reviewer for the comment, we have now explicitly stated that "BP control declined from 68% when defined using a single recent reading to 52% when defined using the two visits' readings" in the abstract	2	37-39
	Comment 2	Clarify the interval between the two BP readings and whether there was heterogeneity across states/facilities.	We have added the intervals between the two reading and have also included a supplementary table 2.	10	232-234
**Reviewer 1**	Comment 3	Specify if missing data handling (e.g., patients without BP measurement in the quarter) could bias the results.	We thank the reviewers to the comments and in our study though some patients had a missed visit in the reporting quarter, which could bias the results, but the age and gender distribution were similar between those with and without missed visits, this is well-represented represented by the large sample. We have addressed this is the limitation and have also added a supplementary table: 4	23	427-428
	Comment 4	While state-level outcomes are shown, a more explicit link with NITI Aayog Health Index scores in the Results section (not only Discussion) would enrich interpretation.	As data were not available for all states, we were unable to include these details in the Results section. Additionally, since the NITI Aayog indices do not include hypertension control indicators, a direct comparison could not be made. However, we have made a detailed interpretation in the discussion section.	18-19	307-326
	**Reviewers**	**Comments to the Author:**	**Revision**	**Page no.**	**line no.**
	Comment 5	Figures: The bar charts are informative, but the y-axis labels in Figure 1 are somewhat cluttered; consider simplifying for clarity.	The suggested changes are incorporated to figure 1.		
	Comment 6	The finding of higher uncontrolled BP in higher-level facilities deserves more explanation—whether due to referral of more severe/complex cases or system-level issues.	We thank the reviewer for the comment and agree with the reviewers that the possible reasons for uncontrolled BP in higher-level could be due the likelihood of complex cases which are being referred and inability to recall the patients who did not visit regularly due	21	388-392

			to lack of outreach community health workers. This has been addressed in the discussion		
	Comment 7	Consider briefly comparing with private sector control rates, as many hypertensives in India seek care outside the public system.	We have added a comparison of government and private sector control for hypertension in India in the discussion section	22-23	408-420
	Comment 8	Expand on policy implications: how can IHCI bridge state disparities, especially in Bihar, Jharkhand, Nagaland?	We have addressed this in the discussion sections	18-19	309-326
	Comment 9	Prescription data absence is acknowledged, but variability in digital recording/reporting quality across states should also be noted.	We have acknowledged this in the limitation that “though all states used simple app for data entry, there could have been differences in the quality of data recording across states.”	23	426-427
	Comment 10	Selection bias: only 5% of estimated hypertensives were enrolled—this may limit generalizability.	We acknowledge the concern regarding potential selection bias due to enrolment of only ~5% of the estimated hypertensive population. However, all enrolled participants were already on treatment, whereas national treatment coverage in India is 14.5% (NNMS). Therefore, while the cohort represents a small subset of all hypertensives, it reflects outcomes among treated patients. We have clarified in the manuscript.	23	430-432
	**Reviewers**	**Comments to the Author:**	**Revision**	**Page no.**	**line no.**
	Comment 11	The manuscript is well written; a few long sentences could be shortened for readability.	We have revised the manuscript, shortening several long sentences to enhance clarity and readability.		

**Reviewer: 2**	Comment 1	It is stated that hypertension was diagnosed according to state-specific protocols, but do the diagnostic criteria for hypertension differ by state? Aren't they standardized as 140/90 mmHg or above? If they are not standardized at 140/90 mmHg, please specify the diagnostic criteria for hypertension for each state.	We thank the reviewer for highlighting this point. We clarify that individuals who had a diagnosis according to state-specific protocols or were already receiving antihypertensive treatment were enrolled. While the diagnostic threshold for blood pressure was systolic ≥140 mmHg or diastolic ≥90 mmHg, treatment protocols varied across states. This clarification has been incorporated into the Methods section	6	129-131
**Reviewer: 2**	Comment 2	Kindly provide a description of what 'Metabases' is and what its primary functions are. Is this application the default option used during visits to medical facilities in India? If not, it is possible that individuals who use digital devices are more health-conscious, which may introduce a potential healthy-user bias. Please consider and discuss this point in the Limitations section.	For this study, we analysed data from all IHCI-implementing facilities, where patient information was entered using a handheld mobile application called Simple. This system was designed to facilitate rapid and accurate patient registration, longitudinal blood pressure recording, and documentation of medication regimens. De-identified data were then extracted using Metabase, an open-source data visualization and analytics platform. We have address this in the Methods sections	8	171-175
	**Reviewers**	**Comments to the Author:**	**Revision**	**Page no.**	**line no.**
	Comment 3	As shown in Figure 1, treatment outcomes among individuals with hypertension vary considerably across states. What are the potential reasons for these disparities? Furthermore, what policy interventions or strategies could be effective in narrowing these gaps? A more in-depth discussion on these issues is encouraged.	We addressed the possible reasons for disparities across states, policy implications and strategies in the discussion sections	18	309-326

Reviewer: 2	Comment 4	In Table 3, younger age is also identified as a significant factor associated with uncontrolled blood pressure. However, this aspect has not been addressed in the Discussion. Poor blood pressure control in younger individuals poses a major risk for the development of cardiovascular complications. Please also discuss the possible reasons for poor blood pressure control among younger adults, as well as potential strategies to address this issue.	We have addressed this in the Discussion by highlighting that younger age was associated with uncontrolled blood pressure, discussing possible reasons for poor control among younger adults, and outlining potential strategies to improve management.	20-21	364-379
	**Reviewers**	**Comments to the Author:**	**Revision**	**Page no.**	**line no.**
	Comment 5	What factors might contribute to better blood pressure control among patients in HWC/SC settings? For instance, could patient education play a significant role? This point should be further discussed.	We addressed the possible reasons for better blood pressure control in HWC/SC in the discussion section with references.	22	402-407
	Comment 6	The sentences are duplicated. Please delete them.	We thank the reviewer for highlighting this, we have now removed the duplicate sentence.	4	88-89
	Comment 7	Please briefly explain the rationale for comparing blood pressure control rates based on a single measurement versus two repeated measurements.	A rationale in the Introduction for comparing blood pressure control rates based on a single measurement versus two repeated measurements, supported by relevant references has been added in the introduction section.	4	91-96
	Comment 8	Please provide the definitions of General Hospital, District Hospital, and Sub-District Hospital in India.	We have elaborated and defined on General Hospital, District Hospital, and Sub-District Hospital using references in the methods sections	7	158-164
	Comment 9	Kindly include the average age of all subjects.	We thank the reviewers for the comment, we have added this in the methods sections	10	223-225

VERSION 2 - REVIEW

**Reviewer**	**2**
**Name**	**Goto, Kenichi**
**Affiliation**	**Kyushu University, Health Sciences**
**Date**	**25-Oct-2025**
**COI**	

The authors have satisfactorily addressed the reviewer's comments
